# LASSIM—A network inference toolbox for genome-wide mechanistic modeling

**DOI:** 10.1371/journal.pcbi.1005608

**Published:** 2017-06-22

**Authors:** Rasmus Magnusson, Guido Pio Mariotti, Mattias Köpsén, William Lövfors, Danuta R. Gawel, Rebecka Jörnsten, Jörg Linde, Torbjörn E. M. Nordling, Elin Nyman, Sylvie Schulze, Colm E. Nestor, Huan Zhang, Gunnar Cedersund, Mikael Benson, Andreas Tjärnberg, Mika Gustafsson

**Affiliations:** 1 Bioinformatics Unit, Department of Physics, Chemistry and Biology, Linköping University, Linköping, Sweden; 2 Centre for Personalised Medicine, Department of Clinical and Experimental Medicine, Linköping University, Linköping, Sweden; 3 Integrative Systems Biology, Department of Biomedical Engineering, Linköping University, Linköping, Sweden; 4 Mathematical Sciences, Chalmers University of Technology, University of Gothenburg, Gothenburg, Sweden; 5 Leibniz-Institute for Natural Product Research and Infection Biology, Hans-Knoell-Institute, Research Group Systems Biology and Bioinformatics, Jena, Germany; 6 Research Group PiDOMICS, Leibniz Institute for Natural Product Research and Infection Biology -Hans Knöll Institute, Jena, Germany; 7 Department of Mechanical Engineering, National Cheng Kung University, Tainan, Taiwan; 8 Stockholm Bioinformatics Center, Science for Life Laboratory, Solna, Sweden; 9 Cell Biology, Department of Clinical and Experimental Medicine, Linköping University, Linköping, Sweden; Max-Planck-Institut für Informatik, GERMANY

## Abstract

Recent technological advancements have made time-resolved, quantitative, multi-omics data available for many model systems, which could be integrated for systems pharmacokinetic use. Here, we present large-scale simulation modeling (LASSIM), which is a novel mathematical tool for performing large-scale inference using mechanistically defined ordinary differential equations (ODE) for gene regulatory networks (GRNs). LASSIM integrates structural knowledge about regulatory interactions and non-linear equations with multiple steady state and dynamic response expression datasets. The rationale behind LASSIM is that biological GRNs can be simplified using a limited subset of core genes that are assumed to regulate all other gene transcription events in the network. The LASSIM method is implemented as a general-purpose toolbox using the PyGMO Python package to make the most of multicore computers and high performance clusters, and is available at https://gitlab.com/Gustafsson-lab/lassim. As a method, LASSIM works in two steps, where it first infers a non-linear ODE system of the pre-specified core gene expression. Second, LASSIM in parallel optimizes the parameters that model the regulation of peripheral genes by core system genes. We showed the usefulness of this method by applying LASSIM to infer a large-scale non-linear model of naïve Th2 cell differentiation, made possible by integrating Th2 specific bindings, time-series together with six public and six novel siRNA-mediated knock-down experiments. ChIP-seq showed significant overlap for all tested transcription factors. Next, we performed novel time-series measurements of total T-cells during differentiation towards Th2 and verified that our LASSIM model could monitor those data significantly better than comparable models that used the same Th2 bindings. In summary, the LASSIM toolbox opens the door to a new type of model-based data analysis that combines the strengths of reliable mechanistic models with truly systems-level data. We demonstrate the power of this approach by inferring a mechanistically motivated, genome-wide model of the Th2 transcription regulatory system, which plays an important role in several immune related diseases.

## Author summary

There are excellent methods to mathematically model time-resolved biological data on a small scale using accurate mechanistic models. Despite the rapidly increasing availability of such data, mechanistic models have not been applied on a genome-wide level due to excessive runtimes and the non-identifiability of model parameters. However, genome-wide, mechanistic models could potentially answer key clinical questions, such as finding the best drug combinations to induce an expression change from a disease to a healthy state. We present LASSIM, which is a toolbox built to infer parameters within mechanistic models on a genomic scale. This is made possible due to a property shared across biological systems, namely the existence of a subset of master regulators, here denoted the *core* system. The introduction of a core system of genes simplifies the network inference into small solvable sub-problems, and implies that all main regulatory actions on *peripheral genes* come from a small set of regulator genes. This separation allows substantial parts of computations to be solved in parallel, i.e. permitting the use of a computer cluster, which substantially reduces computation time.

This is a PLoS Computational Biology methods paper.

## Introduction

The ‘omics’ era of molecular biology has generated enormous amounts of potentially informative molecular data. However, without new methods for data analysis, this tends to drown researchers in data, rather than provide clear and useful insights. Such methods are considered within systems biology, in particular via its usage of mathematical models [1, 2]. This usage includes mathematical models for predictions of the cellular response to different stimuli, such as a change in environmental conditions or the presence of pathogens. Stimuli are typically recognized by cell surface proteins that induce an intracellular signal transmission via signaling cascades to the cell nuclei. Consequently, transcription factors (TFs) bind to their respective target genes which results in a change of gene expression [3]. This is reflected by changes in the cellular transcriptome, which may be described mathematically by gene regulatory networks (GRNs) [4]. GRNs consist of nodes representing genes, and edges, representing gene interactions or more general influences [5].

Scientific progress is hindered by the fact that systems biology currently is divided into large-scale and small-scale modeling subfields; the large-scale modeling approach derives data-driven networks directly from ‘omics’ data, while the small-scale approach performs *non-linear modeling* using mechanistically derived models for specific biological sub-systems. Within the large-scale approach, it is common to handle thousands of unknown parameters, aiming at generating a coarse-grained genome-wide view [6–8]. These types of approaches have been shown to perform relatively well in several benchmark studies [9, 10], examples of approaches being the LASSO [11–13], ARACNE [14], and the Inferelator [13]. However, these methods do not provide a simulation model that can be used to predict new experiments, and individual model parameters might not be reliable. On the other hand, the *mechanistic modeling* approach typically starts with *a priori* formulated hypotheses regarding a relatively small sub-system representing coupled states. These hypotheses are then formulated into equations, typically non-linear ODEs with unknown parameters that are estimated from experimental data. Hypotheses that disagree with data are rejected, and non-rejected hypotheses are used to predict potentially new regulatory interactions to be tested within new experiments [15–22]. However, mechanistic modeling approaches are greatly limited in size since the simulation-derived parameter estimations scale badly with model size [23]. Because of these inherent limitations in both subfields of systems biology, there are today no methods of realistically predicting system-wide changes in biological systems. For instance, these systems i) are highly non-linear, ii) include critical modeling motifs such as feed-forward and feedback regulations, and iii) eventually lead to genome-wide expression changes. Here we present the LArge-Scale SIMulation-based network identification (LASSIM)–a new reverse engineering method of biological systems that resolves i)-iii).

We implemented LASSIM as a toolbox that can handle non-linear ODE modeling of genome-wide processes. The rationale behind LASSIM is that only a subset of all regulators has complex interactions using integrated feedback and feed-forward network motifs with critical internal regulations, while the vast majority of genes are a result of these regulators activity [24]. Moreover, we hypothesize that these regulators can be pre-defined through prior knowledge, i.e. the biological understanding of the system, and these regulators are referred to throughout the article as the *core* system of the specific function. The parameters of the core system can firstly be identified using standard numerical ODE solvers. Once the core system is modeled, the control of peripheral genes can be solved independently. This parallelization of the second part of the network identification enables the run time of LASSIM to linearly scale with the size of the entire genome, which is inspired by the decoupling used in the LASSO method [25]. We have implemented LASSIM as an open source modular Python package using optimized compiled code for computationally heavy algorithmic components. This makes LASSIM fast and flexible enough to handle a great variety of mechanistic and grey-box models.

To show the potential of LASSIM as a flexible and powerful inference tool that can describe genome-wide changes from key pathways, we applied it to identify a minimal robust non-linear model from three kinds of asymmetric data from human Th2 differentiation. This is an ideal model system, since prior work by us and others has shown that the system is transcriptionally controlled by a few relatively well-studied master TFs that orchestrate the expression of a majority of genes [26–31], and that Th2 cells play key roles in regulating the human immune system. We used 12 core TF regulators and, based on DNAase hypersensitivity, we performed binding predictions of the regulatory regions available in Th2 cells, which represented our set of putative interactions. We then applied LASSIM, which performed data fitting and model complexity reduction using time-series gene expression and novel siRNA mediated knock-down experiments during Th2 differentiation such that each TF was directly perturbed once. We assessed the pruned model from randomized subsets of the DNAase predictions in two steps. First, we analyzed the inferred topology of the core system and found that our predicted interactions were significantly better at modeling new Th2 data than other models from the putative interaction list (P<0.006). Second, we analyzed the core-to-gene interactions and found that they were both supported by 14 public ChIP-seq experiments, and identified an enriched set of 685 target genes that had lower cost than 95% of the random models (expected was 385, binomial P<10^−46^). In other words, LASSIM can create non-linear minimal models that span the entire genome, and the example presented herein was shown to predict both new data and network structure correctly.

## Results

### LASSIM infers networks from user input

**Fig 1:**
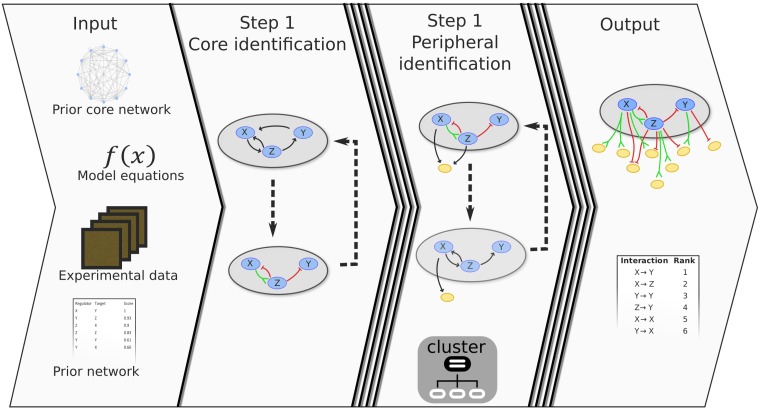
General workflow of the LArge-Scale SIMulation (LASSIM) high performance toolbox. As **Input** LASSIM take four basic components, *core and peripheral prior networks* (optional), *experimental data* and *dynamic equations* to fit a large-scale non-linear dynamic system based on the fully parallel PyGMO toolbox. **Step 1**, LASSIM performs pruning and data fitting on the core system. **Step 2**, LASSIM expands the core model by inferring outgoing interactions with peripheral genes, with each gene solved in parallel using a computer cluster. The LASSIM functions are fully modular, and have been built so that the functions describing the optimization procedure, dynamic equations, cost function and data pruning are modular and can easily be changed by the user. The **Output** is either the core network defined in **Step 1** or a genome wide regulatory network if **Step 2** is run, with ranked interactions based on selection order, as well as kinetic parameters for the dynamic equations.

LASSIM is a newly developed method for large-scale flexible non-linear ODE modeling, with an accompanying implementation built as a Python open source software package (presented in Fig 1), and can be found at https://gitlab.com/Gustafsson-lab/lassim. The rationale behind LASSIM is the empirical observation that many biological processes are controlled by few (less than 50) interlinked regulators that includes feedbacks, hereafter referred to as the *core system*. LASSIM is built to first infer the core system and thereafter solve the regulation of peripheral genes in parallel. This approach significantly reduces the computation time (S1 Fig). Furthermore, LASSIM has been implemented modularly, allowing for methods and algorithms to be easily exchanged, and is built on PyGMO, which is the European Space Agency platform for performing parallel computations of optimization tasks [32].

LASSIM takes as user input the *a priori* structure of a system, dynamic equations, model selection criteria, expression training data (time-series and/or perturbation data), and a confidence interaction list (Fig 1, Input). The default kinetic equations are built on a sigmoid activation component and a linear degradation component according to the results presented in [33, 34]. However, linear, Hill, mass-action, or other user defined kinetics can easily be incorporated in the LASSIM interface by replacing default computational components with kinetics coded as Python functions. The models are by default tuned via backward selection and by minimization of likelihood ratio -tests. LASSIM starts by inferring parameter values for the internal regulations within the core system (Fig 1, Step 1), and unnecessary parameters in the core system are removed using training data, such as response to perturbations and time-series measurements. In a second step, the core-to-peripheral gene regulations are modeled independently, taking advantage of the parallel nature of the problem, again with model selection based on a user-defined criterion (Fig 1, Step 2). Finally, as made possible due to their independence, the combined models are assembled into one single full-scale non-linear ODE model (Fig 1, Output). We used two data generated examples to illustrate that including realistic dynamics provided both improved network structure identification and trajectory modeling compared to a pure static regression-based LASSO approach (S2 Fig).

### LASSIM models Th2 differentiation of naïve T-cells

**Fig 2:**
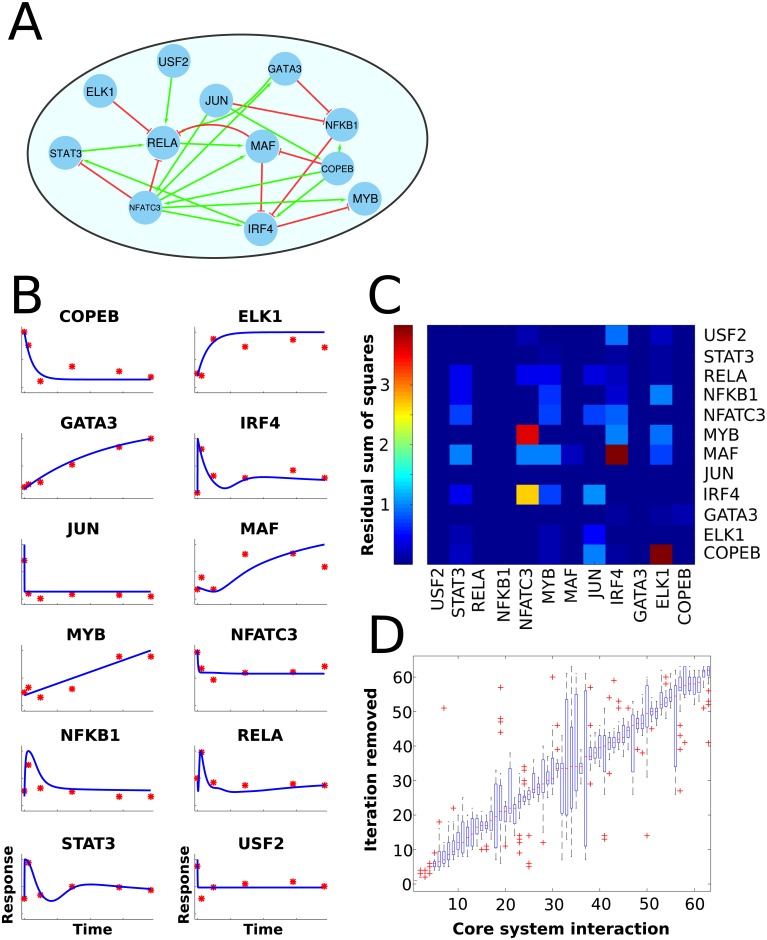
LASSIM inferred a robust minimal and full-scale non-linear transcription factor—Target dynamic system describing naïve T-cells towards Th2 cells. (A) We identified 12 core Th2 driving TFs from the literature, and inferred their putative targets using DNase-seq data from ENCODE. In total, these interactions constituted of 63 core TF-TF interactions and 64,872 core-to-peripheral gene regulations. These interactions were assumed to follow a sigmoid function, as described in the Methods section. The complete prior network was, together with Th2 differentiation dynamics and siRNA mediated knock down data of each TF measured by microarray profiling, used by LASSIM to infer a Th2 core system. As can be seen in the core network, there are feedback loops between several of the TFs. (B) Microarray time series experiments (red dots) and respective state simulated by the LASSIM model (blue solid lines) of the core TFs. On the x-axis is time, and the y-axis denotes gene expression in arbitrary units. (C) Heat map of the data fit of the Th2 model to the siRNA perturbation data, i.e. the siRNA part of *V*(p*). Each siRNA knock-down experiment is represented as a separate column. For example, the model fits the response of a siRNA knock-down on IRF4 well for all TFs except MAF well. (D) Box-plot representing the ranking of each removed parameter from multiple stochastic optimizations of the core model. All edges that had a median selection rank over 40 were included in the final model. Model selection was based on prediction error variation, see section *model selection* and S3 Fig.

**Fig 3:**
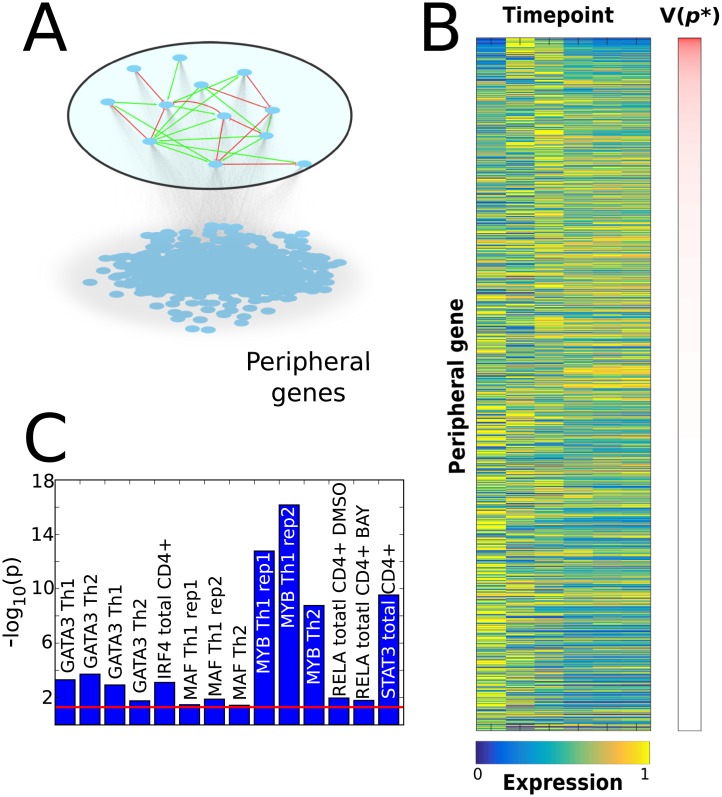
LASSIM inferred a genome-wide model of 35,900 core-to-peripheral gene interactions. (A) These peripheral genes do not have any feedbacks to the core system, nor any crosstalk among themselves. (B) The measured mRNA profiles of the 10,543 peripheral genes (blue/yellow represent relative low/high expression), sorted by the model cost (*V*(p*)) on the y-axis, and time points on the x-axis. As can be seen, genes that display a peak in expression at the second or third time points are generally associated with a higher cost. (C) The results from the ChIP-seq analysis of inferred to-gene interactions, where the y-axis shows the—log_10_(p). The red line denotes the significance level (Bootstrap P < 0.05).

We next applied LASSIM to infer a genome-wide mechanistically motivated model of TF-target relationships of human differentiation of naïve CD4+ T-cells becoming effector Th2 cells, which play a pivotal role in multiple immune-related processes [35]. We first identified a putative core set of 12 TFs (COPEB, ETS1, GATA3, IRF4, JUN, MAF, MYB, NFATC3, NFKB1, RELA, STAT3, USF2) from our previous experimental studies of TFs within the context of Th2 differentiation [35, 36], and a set of 11,083 differentially expressed genes in fully developed Th2 cells vs naïve T-cells (FDR<0.05, see Methods). Second, we predicted 63 core TF-TF putative interactions and 64,872 putative core-to-peripheral interactions with Th2-specific DNase-seq footprints using the HINT bias correction method [37, 38], together with motif matching from the three TF binding motif databases UniProbe, JASPAR and HOCOMOCO using the regulatory genomics toolbox Python package rgt-gen [39–41]. We then trained and pruned the core model in LASSIM (Fig 2A), using our previous microarray time-series data (Fig 2B) from naïve T-cells differentiating into Th2 cells [35], and a compendium of siRNA mediated knock-downs under Th2 differentiation (within 16–24 h) of each of the respective TFs analyzed with microarrays (Fig 2C). We compiled the compendium re-using six previously published siRNA perturbations and created six new ones (Methods) together with 18 data points (each series repeated 3–4 times), for which we applied LASSIM. The initial core system comprised of 216 data points and 87 parameters, for which we applied LASSIM to perform a fitting of the core system to the time-series data and all siRNA knock-down data simultaneously (Fig 2A–2D). The corresponding starting peripheral systems had 18 data points and about eight parameters per gene (Fig 3A).

### Robustness analysis identifies a LASSIM model with a minimal number of parameters and low error

To estimate the robustness of our fitted model we repeated the full LASSIM procedure, including stepwise removal of edges in the core system, 100 times using different starting points and small perturbations of the data (Fig 2D, S3 and S4 Figs). We found robust minimal solutions at 40 removed parameters, simultaneously adhering to the following characteristics: 1) Good fit to the time-series data, i.e. non-rejected solution from a Gaussian noise distribution (Eq 3 in Methods gives cost function *V*(p*) = 15.0, χ^2^_0.95_ (df = 12*6) = 92.8) (Fig 2B). 2) Good fit to the siRNA data (Fig 2C). 3) Robust sets of interactions, i.e. when LASSIM was applied to the same training data starting from different initial parameter sets the orders of parameter removal were highly similar (Fig 2D).

Model selection and parameter estimation is a complex optimization problem due to the large number of variables, the non-convex nature of the objective function, and noisy measurements. We therefore analyzed the sensitivity of the core system, and found the robustness to decrease through bootstrap re-sampling compared to re-runs of LASSIM on the original data (S3 Fig), and that removal of all siRNA data increased the model variance (S4 Fig). These two results supported the system with 39 removed parameters, i.e. the 24 remaining interactions, as a robust minimal core model.

### LASSIM identifies peripheral interactions that are functionally supported by ChIP-seq

Next, LASSIM stepwise pruned the interactions and fitted the parameters representing the core regulation onto the peripheral genes. There was a good agreement between model simulations and time-series measurements (i.e. V(p*) < χ^2^_0.95_ (df = 6) = 12.6) for 10,546 genes (Fig 3B), as well as for the 12 siRNA knock-downs (Fig 2C). The resulting genome-wide model consisted of 35,900 core-to-peripheral gene interactions, thus removing 28,972 potential interactions. To evaluate the performance of LASSIM we tested core-to-peripheral gene edges in two ways. First, we tested the ability of the algorithm to predict data, and found that LASSIM was better at predicting hidden points that linear interpolations between time points (S5 Fig). Second, we examined whether the removed or remaining interactions were more likely to have a physical representation in experimental data. This was done for each individual TF by comparing the mean peak scores of the removed and remaining interactions respectively using ChIP-seq and ChIP-Chip from 14 experiments of five different TFs. [35, 42–44]. We found consistent enrichments for the remaining interactions (permutation test P<0.05) for each experiment, with the highest enrichments from the STAT3 and MYB bindings (P<10^−9^, see Fig 3C). Thus, we feel confident that LASSIM robustly removes interactions that are less likely to be direct than the remaining counterparts.

### LASSIM monitors total CD4+ T-cell dynamics significantly better than other models from the prior network

**Fig 4:**
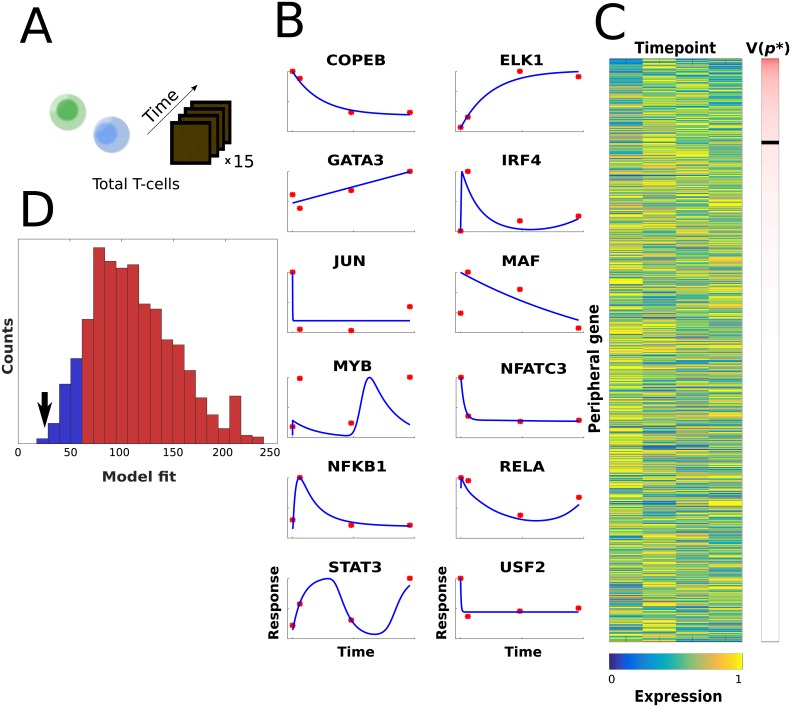
The edges from the naïve Th2 model were better refitted to total Th2 differentiation than the prior network. (A) We refitted the core network to new time-series data from total Th2-cells by keeping the signs of the inferred minimal Th2 model. (B) The fit of the core model to the total T-cell data is shown. The y-axis has arbitrary units of expression, and the x-axis is time. The model output is denoted by the blue curves, and the data are shown by the red points. (C) The fit of the peripheral genes. The figure follows the same style as in Fig 3B. The black band in the cost bar denotes the 0.95 rejection limit of the corresponding χ^2^-test, with all rejected models above the band. (D) We resampled our prior matrix with the same number of parameters and signs as the Th2 model 10,000 times, and compared the random models with the output from LASSIM. The ability of the inferred core network structure to fit novel gene expression data of total T-activation was considered. A total of 1 000 random core model structures were drawn from the prior network, and refitted to the data. The distribution of the fit is shown by the blue curve. The core network identified from LASSIM was shown to be significantly better for fitting the novel data, as marked by the black arrow. Moreover, most of the core models could be rejected by a χ^2^-test, and are represented in red.

To test the general applicability of the inferred Th2 dynamic model, we aimed to test its capability to model Th2 differentiation from a slightly different starting point. Th2 cells were therefore differentiated *in vitro* starting with a mix of naïve, memory and primary CD4+ T-cells, hereafter called the *total* T-cell population, which through their respective cytokine secretion influenced the process. We first asked whether the model developed for Th2 differentiation of naïve CD4+ T-cells could also monitor that of total T-cells. For this purpose, we performed new time-series experiments of four time-points and 15 microarrays (Methods). We reasoned that as the new cell-type also contained memory cells (Fig 4A), the magnitude of the kinetic parameters might change, but we hypothesized that the underlying interactions and signs should be static. We therefore retrained the identified model as well as randomly sampled models from the TF binding prior network and of the same size and parameter sign distribution as the minimal Th2 model. The retraining was performed similarly for all models using the optimization procedure of LASSIM, and our test statistic was the cost function *V(****p****)*. We found a good re-fit for the core system, which passed a χ^2^ -test (*V*(p*) < χ^2^_0.95_(df = 4*12) = 65.2) (Fig 4B) and 9,992 peripheral genes (Fig 4C). Then, we compared the refitted core model against 1,000 null models, which showed that its residual was lower than all except six null models (bootstrap P = 0.006, Fig 4D). To analyze whether these random models represented missed good solutions by LASSIM, we retrained the kinetic parameters of these six models on our first training data, but could not find any random models with as low cost as the identified minimal Th2 model (S6 Fig). Lastly, we proceeded to a similar test for each of our peripheral genes by identifying 100 random models for each gene (i.e. one million random models, S7 Fig). Many peripheral genes had high model complexity both in the random distribution (average in-degree = 5.9) and in the minimal model (average in-degree = 3.4), thus we had low statistical power and could not perform stringent statistical corrections for testing which of the peripheral genes were significantly better than random. However, for 64% of the peripheral genes our model performed better than the average random model drawn from the same prior (binomial test P<10^−130^), and for 685 genes our model performed better than 95% of the corresponding random models (expected 385 genes, P < 10^−46^). In summary, we conclude that the core and peripheral solutions identified by LASSIM can monitor new dynamic data relatively robustly.

## Discussion

Gene regulation constitutes a complex dynamic system that can drive cells to new cell states during differentiation. These systems are often analyzed using mathematical modeling via *mechanistic modeling*, or by large scale statistical and association methods without mechanistic time dependent simulation capabilities. Although these approaches have distinct advantages, few efficient ways of combining them have been identified. Herein, we have presented the LASSIM toolbox, which is built on a small-scale simulation-based framework, and it is therefore theoretically possible to model any combination of standard biochemistry kinetics, including classic mass-action and synergistic Hill-kinetics. LASSIM is built on the assumption that a small-scale *core* model can be found—which is a common approach within the small-scale mechanistic modeling community. The core model is applied as input to the rest of the *peripheral* genes, and this process is computed in parallel, under the assumption that the there are no feedback interactions from the peripheral genes to the core genes. The outcome of LASSIM is a full-scale dynamic gene regulatory model.

At its core, LASSIM is a method for creating such regulatory models, whereas other aspects presented herein, such as the automatized core model construction, should be regarded as supportive functions for facilitating the application of LASSIM. These functions are instead incorporated to allow a user to apply LASSIM with the simplest set of information available. In other words, the only input that is strictly needed for LASSIM is information of what states should form the core model, and experimental data for the complete set. We hypothesize that additional inputs can greatly improve the performance of the method. One such input could be prior networks given by more advanced TF-to-target interaction measurements. For instance, a recent study has introduced a wide array of tissue specific transcriptional networks [45], which could be used as a prior in further implementations of LASSIM.

Although LASSIM is different to other GRN inference methods, it is useful to compare close alternatives. ARACNE is a frequently used fast information theoretical method that produces genome-wide networks [14]. However, it cannot be used to predict the directionality or sign of interaction, and cannot be used to predict the dynamic response upon treatment due to the lack of model equations. Model equations are present in few ODE-based models, such as Inferelator [13], ExTILAR [46], and NetGenerator [47]. Inferelator and ExTILAR are directly based on regression, thus they use estimated time derivatives directly from data to approximate ODEs. ExTILAR [48] uses target genes to infer upstream TF activity and solves a fully coupled regression problem of TF-targets explicitly. After identifying the structure of a model by regression, ExTILAR fits ODEs to that structure to achieve a model, which can be simulated. However, ExTILAR can only infer models of moderate size but not full genomic systems. Inferelator has been built to include great variety of user-inputs, and some different kinetics, while ExTILAR uses linear models. However, neither of the methods can handle mechanistic models with feedbacks. There are several tools for small-scale mechanistic models, where NetGenerator [47] represents one of those commonly used for GRNs. This method is based on linear or non-linear ODEs, and makes use of simulation-based models to estimate parameters. NetGenerator uses a heuristic search strategy with forward selection and backward elimination to infer a sparse network and can, similarly to LASSIM, use several perturbations simultaneously for parameter estimations and model selection. However, this method is not optimized for large-scale systems, and practical examples are limited to some tens of genes. In conclusion, there is to date no other approach that allows for the identification of a GRN simulation model for the entire genome.

The key assumption behind LASSIM is that all genes are affected only by a core system. For the specific example considered here, Th2 differentiation, the studied core genes have already been proposed and studied in previous works [27, 28, 35, 49]. More generally, it is reasonable to assume that the most influential TFs can often be considered as a sub-system, which qualifies as a core system in the LASSIM sense. The two main arguments for this are 1) that as the number of components, i.e. genes, required to sustain a biological function, grows, the easier it becomes to destroy the function [24], and 2) that the interactions within the TF core system can be considered as collapsed versions of more extensive pathways. 1) can be understood by considering that each component works with probability p, then the probability that the function will be performed decreases with the number of components n as p^n^. For instance, if p = 0.99 and n = 10, then the function is performed in 90% of the cases, but if n = 1000, then it is only performed in 0.004% of the cases. A core system with relatively few crucial components should therefore be behind each function. 2) can be understood by considering two hypothetical TFs in a core system: TF_A_ and TF_B_. Assume that TF_A_ affects TF_B_, but via one other gene C outside the core system. In other words, the true pathway is TF_A_ -> C -> TF_B_. In LASSIM, this interaction would still be captured, but as TF_A_ -> TF_B_, i.e. as a collapsed interaction. Such collapsed interactions form the basis of GRNs as such, since GRNs describe the collapsed effect of a gene directly on another gene, even though this effect may in reality be indirect, occurring via, for instance, phosphorylation of proteins. The existence of a core system is therefore likely to be an effective simplification. Note that, in general, a different core system is behind different biological functions, so it is necessary to take special care when combining data from different biological functions.

The application of LASSIM on Th2 differentiation presented herein is of principal importance since it is the first simulation-based model developed for the entire genome, and integrates three different kinds of data, i.e. prior network estimates, time-series, and knock-down data to infer a robust minimal model with realistic dynamics. The first principal requirement was a core system, where we relied on our previous findings such that a majority of core genes originated from Bruhn et al [49], which is a practical example of how a core system could be *a priori* identified. They identified a Th2 module and its upstream TF regulators, where we considered the regulators as core genes. We applied LASSIM to infer a simulation based model of this system, based on Th2-specific binding predictions. Our analysis showed that about 45% of the TF-target predictions could be removed from the model with a similar fit to data. Importantly, the removed interactions were also the ones with the lowest experimental support from ChIP analyses of the individual TFs, which shows support for the inferred peripheral gene regulations. We also tested whether the model was capable of modeling new expression dynamics from mixed cell differentiation. For that purpose, we had to adjust the magnitude of the kinetic parameters. We therefore compared the LASSIM-inferred model against the random models sampled from the TF-target prior predictions and allowed for a similar retraining as for the Th2 model. We found both that the core model and an enriched fraction of the peripheral genes were significantly better modeled by LASSIM. Interestingly, the core TFs MAF and MYB yielded the worst fit to the dynamic curves, but still significantly overlapped with ChIP-seq (P_MAF_ <0.04, P_MYB_ <2*10^−9^). These observations suggest that our modeled dynamics might reflect activation from post-translational modifications of MAF and MYB, where, for example, tyrosine phosphorylation of c-MAF has been shown to increase IL4 and thereby amplify Th2 differentiation in mice [50]. From an experimental design point of view, the LASSIM output also demonstrated the need for perturbation data of all regulators in the studied core system to have enough information in data for making model claims. Moreover, by analyzing the underlying motifs in the core model, we can observe feed-forward and feedback loops, e.g. the feedback STAT3-RELA-MAF-IRF4-STAT3 path. Hence, ODE models are needed to perform pharmacokinetic modeling of drugs. In summary, LASSIM enables the large-scale modeling of several data types including knock-downs and phosphorylation time-series, for which mechanistic non-linear models are needed, for example to include mass-preserving constraints.

## Methods

### Selection of core system TFs and predicted interactions

Several TFs have been tested for being associated with Th2 differentiation. We base our selection on the following four studies: 1) In Bruhn et al. [49], we performed siRNA of 25 TFs and found seven highly relevant (GATA3, MAF NFATC3, STAT5A, STAT3, NFKB1, JUN). 2) In Gustafsson et al. [35] we also identified the TF MYB as having great importance to Th2. 3) We have also identified IRF4 and ETS1 as key TFs of Th2 differentiation [43]. 4) By analyzing data from Nestor *et al* [36] we identified USF2, and KLF6 as potentially interesting Th2 TFs as they were differentially expressed in unstimulated non-symptomatic allergic patients compared to the expression from controls (P_USF2_ = 3 * 10^−9^, P_KLF6_ = 0.001).

### Model equations

Systems of ordinary differential equations ODEs are frequently employed in engineering to mathematically model the states of a system. Let the *i*:th state be denoted *x*_*i*_, and let all states be collected in a vector ***x***. The dynamics of the states are given by ODEs (Eq 1), which describe the changes using a function *f* of states ***x*** and additional parameters ***p***.

$$\frac{dx}{dt}\left(t\right)=f\left(x,p\right)$$ (1)

Simulation-based approaches, like LASSIM, can be developed for any form of *f*, e.g. non-linear expressions such as Michaelis-Menten or mass-action expressions. For gene regulation mechanisms, however, a more suitable form of *f* is a sigmoid regulation, used in e.g. [33, 34], as shown in Eq (2):

$$\frac{dx_{i}}{dt}\left(t\right)=-\lambda_{i}x_{i}+\frac{\xi_{i}}{1+e^{-\sum_{j}w_{i,j}x_{j}}}$$ (2)

In Eq (2), the degradation rate of gene *x*_*i*_ is negatively dependent on the concentration, while the regulators *x*_*j*_ can have both inhibitory and enhancing effects. The rate constants are *λ*_*i*_, ξ_*i*_, which are strictly positive, and *w*_*ij*_, which can be both negative and positive. Given this equation, the value of the second term is restricted between 0 and ξ_*i*_ and the equation system is stable for all *λ*_*i*_, ξ_*i*_ *ϵ*]0, ∞[. The search bounds of the parameters were defined to be *λ*_*i*_, ξ_*i*_ *ϵ* [0, 20], *w*_*ij*_ *ϵ* [−20, 20] which covers almost all possible values of the term (S8 Fig).

### Model selection

When fitting the model to data, the objective function was set to minimize the sum of squares.

$$V\left(p^{*}\right)={\text{min}}_{p}\sum_{i,j}\frac{{\left(y_{i,j}-{\hat{y}}_{i,j}\left(p\right)\right)}^{2}}{\sigma_{i,j}{}^{2}}$$ (3)

In Eq (3), the best parameter vector, p*, is the parameter set that minimizes the difference of the measurements y and model output $\hat{y}$ for state *i* and time point *j*. For the knock-down data, the fit was calculated correspondingly, but with the standard deviation σ set to 1 due to data scarcity. This selection of σ also made the cost contributions *V(****p****)* from the knock down and time simulations to be of the same order of magnitude for most parameter sets. For the time series data, standard deviations were estimated using all time points. Both the perturbation and time series model output were fitted using Eq (3). Moreover, the structure of Eq (3) can be used to fit any type of model output, for instance, to model dose-response curves, gene knock-in experiments, or steady state data.

The model selection process in LASSIM can use any of the standard tools, such as minimizing the Akaike Information Criteria (AIC), Bayesian Information Criteria (BIC), cross-validation error, or by utilizing *χ*^2^-tests [21]. In the Th2 model, our inclusion of the siRNA perturbations made the theoretical considerations unable to meet the criteria for the AIC, as the cost *V*(**p**) was a function of data points with and without the possibility of estimated standard deviation. We instead used a heuristic criterion to find a balance between model fit and sparsity, where the first edge removal that yielded a cost increase larger than 0.05 times the cost standard deviation of the parameter sets was chosen. The parameter value of 0.05 was chosen after studying the core system behavior, and deciding to correspond to the sparsest model with a retained fit of the data (S3 Fig, 38 parameters removed). To evaluate the fit between model and unperturbed time series data, we used χ^2^-tests [21]. In the χ^2^-tests, the degrees of freedom were set to equal the number of data points, and the rejection limit was set to α = 0.05.

### Bootstrap of optimization and model selection sensitivity

The minimization of Eq (3) is a complex problem, and the risk of optimization failure is large, Moreover, optimization has a stochastic element, and the outcome varies. We therefore tested whether optimization failure had a greater impact on the LASSIM outcome than the information embedded in the data. We performed 24 corresponding bootstrap experiments, where individual time-series- and knock down data points were assigned a homogenously distributed cost weight *τ ϵ* [0, 1], as seen in Eq 4.

$$V_{bootstrap}\left(p^{*}\right)={\text{min}}_{p}\sum_{i,j}\tau_{i,j}\;\frac{{\left(y_{i,j}-{\hat{y}}_{i,j}\left(p\right)\right)}^{2}}{\sigma_{i,j}{}^{2}}$$ (4)

We found that the robustness of the parameter removal, in terms of removal order and final cost V_bootstrap_(p*), was lower than the first comparison (S3 Fig). Even though approximately the same number of interactions could be removed, the chosen parameter set was different between different bootstrap experiments. This fact indicates that phenomena such as optimization failure have a nominal impact on model selection compared to the quality and certainty of our data.

### Time-series data from Th2 polarized total CD4+ T cells

Total CD4+ cells were isolated from PBMCs using isolation kits from Miltenyi (Bergisch Gladbach, Germany) according to the manufacturer’s instruction. The isolated cells were washed, activated, and the Th2 cells were polarized as previously described [35]. Briefly, the isolated total CD4+ cells were activated with plate-bound anti-CD3 (500ng/ml), 500ng/ml soluble anti-CD28, 5μg/ml anti-IL-12, 10ng/ml IL-4 and 17ng/ml IL-2 (R&D Systems). For microarray experiments, 200 ng of total RNA was labeled and transcribed to cRNA using the Quick Amp Labeling Kit (Agilent, USA), then purified using the RNeasy mini kit and hybridized to Agilent Human GE 4x44 K v2 slides, according to the manufacturer’s instructions. Slides were scanned using the Agilent Microarray scanner 2505C, and raw data were obtained using the Feature Extraction Software (Agilent). Moreover, we only considered data for genes that were deemed to respond to the stimulation, such that we could reject the changes of expression over time from being white noise measurements, i.e. reject if the sum of residuals from the null-model < χ^2^_0.95_(df = 4).

### *In vitro* knock-down experiments

In vitro knock-downs were generated from human naïve CD4+ T-cells isolated from healthy donor buffy coats using magnetic bead separation (Miltenyi Biotec, Sweden). Typically, 1x10^6^ cells were transfected with 600nM on-target plus SMART pool against IRF4 (Dharmacon, USA), MAF, GATA3, USF2, COPEB (Thermo Fisher Scientific Inc, USA), or non-targeting siRNA (Dharmacon, USA) using Amaxa transfection (U-014). After six hours of incubation, the cells were washed and subsequently activated with plate-bound anti-CD3 (500 ng/ml), soluble anti-CD28 (500 ng/ml) and polarized towards Th2 using IL-4 (10 ng/ml), IL-2 (17 ng/ml) and anti-IL-12 (5 μg/ml, R&D Systems, USA) for 24 hours for each of the TFs siRNA (12h for IRF4). The cells were then harvested in Qiazol and total RNA was extracted using the RNeasy mini kit (QIAGEN, Germany). Expression was analyzed using the Human GE 4x44K v2 Microarray Kit from Agilent Technologies. All siRNA-induced knock-downs were followed by a short Th2 polarization (24h for all except IRF4, which had 12h) and microarray analysis, and control experiments corresponding Th2 polarization using scrambled non-targeting siRNA.

### *In silico* knock-downs

To overcome possible hurdles with dynamics outside the prediction ability of the model, and thus any bias in model fit, the data was truncated between -2 and 2. The knock-downs can be *in silico* modeled in a great variety of ways, and in LASSIM we chose to model homozygotic and heterozygotic knock-downs by increasing the degradation constant in Eq (2), such that:

$${\tilde{\lambda }}_{i}=\lambda_{i}*\left(\frac{x_{i}}{{\tilde{x}}_{i}}\right)$$ (5)

In Eq 5, the augmented *λ* is estimated from the inverse of the data fold change. For each of the *in silico* siRNA knock-downs, 12 independent simulations were performed. The perturbations were approximated by increasing the self-regulatory term λ for each of the studied TFs. This increase was set to be proportionate to the measured decrease of said knocked gene.

## Data access

The data sets for this study are available at the GEO data repository under super series GSE60683.

## Supporting information

S1 Fig The time scale of the LASSIM algorithm. It was found that the time needed for the search algorithm to find a parameter solution that passes a χ^2^ -test increased exponentially with number of parameters.
(EPS)

S2 Fig LASSIM improved dynamic trajectories and structure compared to LASSO on two computer-generated examples. (A) Simulations using LASSIM (dashed blue line) and LASSO (red solid lines) derived models on a synthetic dataset. The black dots and lines shows sampled and real expression of IRSP over time. LASSIM can identify the overshoot of IRSP between the first and second time-point correctly, whereas LASSO cannot. (B) LASSIM (red dots) receives higher AUC than our previous best performer LASSO method (blue line) in the DREAM2 network identification challenge in terms of precision [25]. LASSIM backward selection using the linear transfer function was applied to all connections with confidence score of >80% from our previous approach, and therefore has no predictions for recall>0.22. The area under the curve (AUC) of the receiver operating curve (ROC) was about 20% higher for LASSIM than for LASSO (P<0.03).
(EPS)

S3 Fig Sensitivity analysis of core model identification. When removing edges from the model, the randomness imbedded in the stochastic optimization will give rise to a difference in the system behavior (blue lines). To test if such optimization artifacts have a larger impact on the model selection than the quality of the data, a sensitivity analysis was performed, where each data point was given a uniformly distributed random weight τ between 0 and 1. The weighted version of the goodness-of-fit as a function of removed edges are plotted in red. As seen, the changes in the data weights induce a higher uncertainty to the core model identification. Moreover, the unperturbed model starts to rise at 40 removed parameters, which was used when determining core model sparsity.
(EPS)

S4 Fig The impact of adding siRNA mediated knock-down data to the core model inference. We tested the benefit of adding the siRNA knock-down data as perturbations in the model inference, and found that the stability of the network inference dramatically increased. On the left side graphs, the model behavior without the perturbation data is shown. The results can be compared with the more stable version on the right, where knock data have been used.
(EPS)

S5 Fig The ability of LASSIM to predict data on peripheral genes. We tested if LASSIM could predict data by drawing >1,700 genes from the naïve Th2 differentiation time series data and randomly hiding a data point from the network inference. We compared the LASSIM prediction with that of a linear interpolation between the prior and subsequent data points, and tested for a difference of residuals using a Wilcoxon signed-rank test. We found that the residuals were slightly lower in the LASSIM results than from the interpolated data (P<0.02) and therefore concluded that LASSIM can accurately predict data. To the left, the figure shows 6 randomly drawn predictions on target genes. In all plots, the x-axis depicts time while the y-axis depicts expression in arbitrary units. The black dot was hidden in the model training. To the right, the performance of three methods for predicting data are shown, where the LASSIM prediction displays the lowest residuals.
(EPS)

S6 Fig The fit of the random core models to the naïve data. The models that were found to fit the total t-cell data better than our prediction, were tested at the original training data, i.e. the naïve t-cell data. It was found that none could fit the data better than the prediction. Nevertheless, none of the models could be rejected using a χ^2^-test.
(EPS)

S7 Fig Distribution of the certainty of the peripheral genes. The distribution of the fits of the peripheral genes on the total T-cell expression time series are shown. Here, the x-axis shows the goodness of fit (as calculated by Eq 3), and the y-axis correspond to the frequencies.
(EPS)

S8 Fig The saturation effect of the input to each gene. The nodes were modeled to be nonlinearly dependent on each other. The figure shows the potential values of the input from other nodes. More specifically, the values are plotted such that they cover the range of the parameter search span for the minimal case of only one input to a transcription factor.
(EPS)

S1 Text Benchmark study using *in silico* DREAM2 challenge. (DOCX)

## References

[1] Kitano H., Systems biology: a brief overview. Science, 2002 295(5560): p. 1662–4. doi: 10.1126/science.1069492 11872829 10.1126/science.1069492

[2] Palsson B., In silico biology through "omics". Nat Biotechnol, 2002 20(7): p. 649–50. doi: 10.1038/nbt0702-649 12089538 10.1038/nbt0702-649

[3] Gonçalves E., et al Bridging the layers: towards integration of signal transduction, regulation and metabolism into mathematical models. Mol Biosyst, 2013 9(7): p. 1576–83. doi: 10.1039/c3mb25489e 23525368 10.1039/c3mb25489e

[4] Linde J., et al Data- and knowledge-based modeling of gene regulatory networks: An update. 2015: EXCLI Journal. 10.17179/excli2015-168 PMC4817425 27047314

[5] de Jong H., Modeling and simulation of genetic regulatory systems: a literature review. J Comput Biol, 2002 9(1): p. 67–103. doi: 10.1089/10665270252833208 11911796 10.1089/10665270252833208

[6] Gustafsson M., Hörnquist M., and Lombardi A., Constructing and analyzing a large-scale gene-to-gene regulatory network—lasso-constrained inference and biological validation. IEEE/ACM Trans Comput Biol Bioinform, 2005 2(3): p. 254–61. doi: 10.1109/TCBB.2005.35 17044188 10.1109/TCBB.2005.35

[7] Gustafsson M., et al Genome-wide system analysis reveals stable yet flexible network dynamics in yeast. IET Syst Biol, 2009 3(4): p. 219–28. doi: 10.1049/iet-syb.2008.0112 19640161 10.1049/iet-syb.2008.0112

[8] Marbach D., et al Revealing strengths and weaknesses of methods for gene network inference. Proc Natl Acad Sci U S A, 2010 107(14): p. 6286–91. doi: 10.1073/pnas.0913357107 20308593 10.1073/pnas.0913357107 PMC2851985

[9] Stolovitzky G., Monroe D., and Califano A., Dialogue on reverse-engineering assessment and methods: the DREAM of high-throughput pathway inference. Ann N Y Acad Sci, 2007 1115: p. 1–22. doi: 10.1196/annals.1407.021 17925349 10.1196/annals.1407.021

[10] Tjarnberg A., et al Avoiding pitfalls in L-1-regularised inference of gene networks. Molecular Biosystems, 2015 11(1): p. 287–296. doi: 10.1039/c4mb00419a 25377664 10.1039/c4mb00419a

[11] Vignes M., et al Gene regulatory network reconstruction using Bayesian networks, the Dantzig Selector, the Lasso and their meta-analysis. PLoS One, 2011 6(12): p. e29165 doi: 10.1371/journal.pone.0029165 22216195 10.1371/journal.pone.0029165 PMC3246469

[12] Menéndez P., et al Gene regulatory networks from multifactorial perturbations using Graphical Lasso: application to the DREAM4 challenge. PLoS One, 2010 5(12): p. e14147 doi: 10.1371/journal.pone.0014147 21188141 10.1371/journal.pone.0014147 PMC3004794

[13] Bonneau R., et al The Inferelator: an algorithm for learning parsimonious regulatory networks from systems-biology data sets de novo. Genome Biol, 2006 7(5): p. R36 doi: 10.1186/gb-2006-7-5-r36 16686963 10.1186/gb-2006-7-5-r36 PMC1779511

[14] Margolin A.A., et al ARACNE: an algorithm for the reconstruction of gene regulatory networks in a mammalian cellular context. BMC Bioinformatics, 2006 7 Suppl 1: p. S7. 10.1186/1471-2105-7-S1-S7 PMC1810318 16723010

[15] Palmér R., et al Effects of IL-1β-Blocking Therapies in Type 2 Diabetes Mellitus: A Quantitative Systems Pharmacology Modeling Approach to Explore Underlying Mechanisms. CPT Pharmacometrics Syst Pharmacol, 2014 3: p. e118 doi: 10.1038/psp.2014.16 24918743 10.1038/psp.2014.16 PMC4076803

[16] Forsgren M.F., et al Physiologically realistic and validated mathematical liver model revels hepatobiliary transfer rates for Gd-EOB-DTPA using human DCE-MRI data. PLoS One, 2014 9(4): p. e95700 doi: 10.1371/journal.pone.0095700 24748411 10.1371/journal.pone.0095700 PMC3991717

[17] Brännmark C., et al Insulin signaling in type 2 diabetes: experimental and modeling analyses reveal mechanisms of insulin resistance in human adipocytes. J Biol Chem, 2013 288(14): p. 9867–80. doi: 10.1074/jbc.M112.432062 23400783 10.1074/jbc.M112.432062 PMC3617287

[18] Nyman E., et al Mechanistic explanations for counter-intuitive phosphorylation dynamics of the insulin receptor and insulin receptor substrate-1 in response to insulin in murine adipocytes. FEBS J, 2012 279(6): p. 987–99. doi: 10.1111/j.1742-4658.2012.08488.x 22248283 10.1111/j.1742-4658.2012.08488.x

[19] Becker V., et al Covering a broad dynamic range: information processing at the erythropoietin receptor. Science, 2010 328(5984): p. 1404–8. doi: 10.1126/science.1184913 20488988 10.1126/science.1184913

[20] Swameye I., et al Identification of nucleocytoplasmic cycling as a remote sensor in cellular signaling by databased modeling. Proc Natl Acad Sci U S A, 2003 100(3): p. 1028–33. doi: 10.1073/pnas.0237333100 12552139 10.1073/pnas.0237333100 PMC298720

[21] Cedersund G. and Roll J., Systems biology: model based evaluation and comparison of potential explanations for given biological data. FEBS J, 2009 276(4): p. 903–22. doi: 10.1111/j.1742-4658.2008.06845.x 19215297 10.1111/j.1742-4658.2008.06845.x

[22] Magnusson R., et al Crosstalks via mTORC2 can explain enhanced activation in response to insulin in diabetic patients. Biosci Rep, 2016. 10.1042/BSR20160514 PMC5271673 27986865

[23] Keogh E. and Mueen A., Curse of Dimensionality, in Encyclopedia of Machine Learning, Sammut C. and Webb G.I., Editors. 2010, Springer US: Boston, MA p. 257–258.

[24] Nordling T.E., et al Deduction of intracellular sub-systems from a topological description of the network. Mol Biosyst, 2007 3(8): p. 523–9. doi: 10.1039/b702142a 17639126 10.1039/b702142a

[25] Gustafsson M., et al Reverse engineering of gene networks with LASSO and nonlinear basis functions. Ann N Y Acad Sci, 2009 1158: p. 265–75. doi: 10.1111/j.1749-6632.2008.03764.x 19348648 10.1111/j.1749-6632.2008.03764.x

[26] Palacios R., et al A network analysis of the human T-cell activation gene network identifies JAGGED1 as a therapeutic target for autoimmune diseases. PLoS One, 2007 2(11): p. e1222 doi: 10.1371/journal.pone.0001222 18030350 10.1371/journal.pone.0001222 PMC2077806

[27] Pedicini M., et al Combining network modeling and gene expression microarray analysis to explore the dynamics of Th1 and Th2 cell regulation. PLoS Comput Biol, 2010 6(12): p. e1001032 doi: 10.1371/journal.pcbi.1001032 21187905 10.1371/journal.pcbi.1001032 PMC3002992

[28] Zhu J. and Paul W.E., Peripheral CD4+ T-cell differentiation regulated by networks of cytokines and transcription factors. Immunol Rev, 2010 238(1): p. 247–62. doi: 10.1111/j.1600-065X.2010.00951.x 20969597 10.1111/j.1600-065X.2010.00951.x PMC2975272

[29] O'Shea J.J., et al Genomic views of STAT function in CD4+ T helper cell differentiation. Nat Rev Immunol, 2011 11(4): p. 239–50. doi: 10.1038/nri2958 21436836 10.1038/nri2958 PMC3070307

[30] Yamane H. and Paul W.E., Early signaling events that underlie fate decisions of naive CD4(+) T cells toward distinct T-helper cell subsets. Immunol Rev, 2013 252(1): p. 12–23. doi: 10.1111/imr.12032 23405892 10.1111/imr.12032 PMC3578301

[31] Christie D. and Zhu J., Transcriptional regulatory networks for CD4 T cell differentiation. Curr Top Microbiol Immunol, 2014 381: p. 125–72. doi: 10.1007/82_2014_372 24839135 10.1007/82_2014_372 PMC4556129

[32] Izzo D., Ruciński M., and Biscani F., The Generalized Island Model, in Parallel Architectures and Bioinspired Algorithms, Fernández de Vega F., Hidalgo Pérez J.I., and Lanchares J., Editors. 2012, Springer Berlin Heidelberg: Berlin, Heidelberg p. 151–169.

[33] Vu T.T. and Vohradsky J., Nonlinear differential equation model for quantification of transcriptional regulation applied to microarray data of Saccharomyces cerevisiae. Nucleic Acids Res, 2007 35(1): p. 279–87. doi: 10.1093/nar/gkl1001 17170011 10.1093/nar/gkl1001 PMC1802551

[34] Yip K.Y., et al Improved reconstruction of in silico gene regulatory networks by integrating knockout and perturbation data. PLoS One, 2010 5(1): p. e8121 doi: 10.1371/journal.pone.0008121 20126643 10.1371/journal.pone.0008121 PMC2811182

[35] Gustafsson M., et al A validated gene regulatory network and GWAS identifies early regulators of T cell-associated diseases. Sci Transl Med, 2015 7(313): p. 313ra178 doi: 10.1126/scitranslmed.aad2722 26560356 10.1126/scitranslmed.aad2722

[36] Nestor C.E., et al DNA methylation changes separate allergic patients from healthy controls and may reflect altered CD4+ T-cell population structure. PLoS Genet, 2014 10(1): p. e1004059 doi: 10.1371/journal.pgen.1004059 24391521 10.1371/journal.pgen.1004059 PMC3879208

[37] Gusmao E.G., et al Analysis of computational footprinting methods for DNase sequencing experiments. Nat Meth, 2016 13(4): p. 303–309. 10.1038/nmeth.3772 26901649

[38] An integrated encyclopedia of DNA elements in the human genome. Nature, 2012 489(7414): p. 57–74. doi: 10.1038/nature11247 22955616 10.1038/nature11247 PMC3439153

[39] Kulakovskiy I.V., et al HOCOMOCO: expansion and enhancement of the collection of transcription factor binding sites models. Nucleic Acids Res, 2016 44(D1): p. D116–25. doi: 10.1093/nar/gkv1249 26586801 10.1093/nar/gkv1249 PMC4702883

[40] Hume M.A., et al UniPROBE, update 2015: new tools and content for the online database of protein-binding microarray data on protein-DNA interactions. Nucleic Acids Res, 2015 43(Database issue): p. D117–22. doi: 10.1093/nar/gku1045 25378322 10.1093/nar/gku1045 PMC4383892

[41] Mathelier A., et al JASPAR 2016: a major expansion and update of the open-access database of transcription factor binding profiles. Nucleic Acids Res, 2016 44(D1): p. D110–5. doi: 10.1093/nar/gkv1176 26531826 10.1093/nar/gkv1176 PMC4702842

[42] Kanhere A., et al T-bet and GATA3 orchestrate Th1 and Th2 differentiation through lineage-specific targeting of distal regulatory elements. Nat Commun, 2012 3: p. 1268 doi: 10.1038/ncomms2260 23232398 10.1038/ncomms2260 PMC3535338

[43] Bruhn S., et al Increased expression of IRF4 and ETS1 in CD4+ cells from patients with intermittent allergic rhinitis. Allergy, 2012 67(1): p. 33–40. doi: 10.1111/j.1398-9995.2011.02707.x 21919915 10.1111/j.1398-9995.2011.02707.x PMC3237963

[44] Durant L., et al Diverse targets of the transcription factor STAT3 contribute to T cell pathogenicity and homeostasis. Immunity, 2010 32(5): p. 605–15. doi: 10.1016/j.immuni.2010.05.003 20493732 10.1016/j.immuni.2010.05.003 PMC3148263

[45] Marbach D., et al Tissue-specific regulatory circuits reveal variable modular perturbations across complex diseases. Nat Methods, 2016 13(4): p. 366–70. doi: 10.1038/nmeth.3799 26950747 10.1038/nmeth.3799 PMC4967716

[46] Hecker M., et al Integrative modeling of transcriptional regulation in response to antirheumatic therapy. BMC Bioinformatics, 2009 10: p. 262 doi: 10.1186/1471-2105-10-262 19703281 10.1186/1471-2105-10-262 PMC2757030

[47] Weber M., et al, Inference of dynamical gene-regulatory networks based on time-resolved multi-stimuli multi-experiment data applying NetGenerator V2.0. BMC Syst Biol, 2013 7: p. 1 doi: 10.1186/1752-0509-7-1 23280066 10.1186/1752-0509-7-1 PMC3605253

[48] Vlaic S., et al, The extended TILAR approach: a novel tool for dynamic modeling of the transcription factor network regulating the adaption to in vitro cultivation of murine hepatocytes. BMC Syst Biol, 2012 6: p. 147 doi: 10.1186/1752-0509-6-147 23190768 10.1186/1752-0509-6-147 PMC3573979

[49] Bruhn S., et al, A generally applicable translational strategy identifies S100A4 as a candidate gene in allergy. Sci Transl Med, 2014 6(218): p. 218ra4 doi: 10.1126/scitranslmed.3007410 24401939 10.1126/scitranslmed.3007410 PMC4539009

[50] Lai C.Y., et al, Tyrosine phosphorylation of c-Maf enhances the expression of IL-4 gene. J Immunol, 2012 189(4): p. 1545–50. doi: 10.4049/jimmunol.1200405 22798672 10.4049/jimmunol.1200405

