# Supplementary material for: LASSIM—A network inference toolbox for genome-wide mechanistic modeling
Source: PLoS Comput Biol. 2017 Jun 22;13(6):e1005608. doi: 10.1371/journal.pcbi.1005608 (PMC5501685; doi:10.1371/journal.pcbi.1005608)
Supplement: S1 Text — (DOCX) [file pcbi.1005608.s009.docx]

**Benchmark study using *in silico* DREAM2 challenge**The DREAM challenges represent an excellent repository with which to benchmark LASSIM. Here we could address the question of whether ODE modeling using LASSIM could lead to higher structural sensitivity than the difference equation based LASSO approach? We choose the DREAM2 Insilico2 challenge as a benchmark test, because it has a similar network structure of many real GRNs and since we previously were selected as best performer for this challenge using the LASSO [25]. Therefore, we had the previously best solution to compare with. The data were generated from an artificial network of 50 genes, where the rate of synthesis of the mRNA of each gene is affected by the level of mRNA of other genes [2]. The data consists of 23 perturbations with 26 time-points each, and in addition, there were heterozygotic and homozygotic knockdowns followed by steady state measurements for each gene. We used the same data pre-processing as the winning LASSO implementation [25]. We applied LASSIM using a linear transfer function and created a prior knowledge matrix such that all interactions with a confidence score of at least 0.8 in our previous LASSO inference, were set to 𝑃=1 and all other interactions were set to 𝑃=0. This restriction corresponded to a system of 103 parameters of 53 interactions and was utilized to decrease the computation time needed for LASSIM to infer the system.

LASSIM identified the parameters of the remaining system and continuously removed one edge at the time until all edges had been removed. The edges were then ranked by the order they had been removed by LASSIM, and the inferred network was then evaluated against the golden standard by calculating precision and recall values for all edges based on their ranks similar to the DREAM assessment. Precision was defined as the fraction of suggested interactions that were true, and recall is defined as the fraction of true interactions that the suggested interactions cover. Similar to DREAM the area under the curve (AUC) of the receiver operating curve (ROC) was used to determine which methods performed best [3] (Fig. S2B). When comparing the AUC results from LASSIM to the previous best performer LASSO on this data [25], we found that AUC increased by 10% (from 0.2563 to 0.2822). Furthermore, comparing the results to the second scoring team which received AUC=0.1549 we see that the increase in AUC score was 41% [4]. However, the comparison between LASSIM and our previous implementation of LASSO should only be performed on the result of the inferred edges where LASSIM was implemented (53 interactions). Then, the AUC increased 20% (from 0.1337 to 0.1598). In order to test if the increase was statistically significant we calculated a null distribution by performing 100,000 random orderings of the 53 interactions we had, thus creating 100,000 random AUCs. We found that 1,920 of the random permutations received a higher AUC than LASSO, for which only 60 also were higher than the LASSIM AUC. In summary, this analysis supported that the dynamical modeling approach increased the structural sensitivity significantly compared to the state-of-the-art LASSO approach (P = 0.03).

# References

25. Gustafsson, M., et al., *Reverse engineering of gene networks with LASSO and nonlinear basis functions.* Ann N Y Acad Sci, 2009. **1158**: p. 265-75.

2. Hoops, S., et al., *COPASI--a COmplex PAthway SImulator.* Bioinformatics, 2006. **22**(24): p. 3067-74.

3. Keilwagen, J., I. Grosse, and J. Grau, *Area under precision-recall curves for weighted and unweighted data.* PLoS One, 2014. **9**(3): p. e92209.

4. Stolovitzky, G., R.J. Prill, and A. Califano, *Lessons from the DREAM2 Challenges.* Ann N Y Acad Sci, 2009. **1158**: p. 159-95.
